# Histaminergic Innervation of the Ventral Anterior Thalamic Nucleus Alleviates Motor Deficits in a 6-OHDA-Induced Rat Model of Parkinson’s Disease

**DOI:** 10.1007/s12264-024-01320-0

**Published:** 2024-12-02

**Authors:** Han-Ting Xu, Xiao-Ya Xi, Shuang Zhou, Yun-Yong Xie, Zhi-San Cui, Bei-Bei Zhang, Shu-Tao Xie, Hong-Zhao Li, Qi-Peng Zhang, Yang Pan, Xiao-Yang Zhang, Jing-Ning Zhu

**Affiliations:** 1 State Key Laboratory of Pharmaceutical Biotechnology, National Resource Center for Mutant Mice, Department of Anesthesiology, Nanjing Drum Tower Hospital, and Department of Physiology, School of Life Sciences, Nanjing University, Nanjing, 210023 China; 2 Institute for Brain Sciences, Nanjing University, Nanjing, 210023 China; 3 Chemistry and Biomedicine Innovation Center (ChemBIC), ChemBioMed Interdisciplinary Research Center, Nanjing University, Nanjing, 210023 China; 4 Department of Geriatric Neurology, Affiliated Brain Hospital of Nanjing Medical University, Nanjing, 210029 China

**Keywords:** Histamine, H1 receptor, H2 receptor, Ventral anterior nucleus of the thalamus, Parkinson’s disease

## Abstract

The ventral anterior (VA) nucleus of the thalamus is a major target of the basal ganglia and is closely associated with the pathogenesis of Parkinson’s disease (PD). Notably, the VA receives direct innervation from the hypothalamic histaminergic system. However, its role in PD remains unknown. Here, we assessed the contribution of histamine to VA neuronal activity and PD motor deficits. Functional magnetic resonance imaging showed reduced VA activity in PD patients. Optogenetic activation of VA neurons or histaminergic afferents significantly alleviated motor deficits in 6-OHDA-induced PD rats. Furthermore, histamine excited VA neurons *via* H1 and H2 receptors and their coupled hyperpolarization-activated cyclic nucleotide-gated channels, inward-rectifier K^+^ channels, or Ca^2+^-activated K^+^ channels. These results demonstrate that histaminergic afferents actively compensate for Parkinsonian motor deficits by biasing VA activity. These findings suggest that targeting VA histamine receptors and downstream ion channels may be a potential therapeutic strategy for PD motor dysfunction.

## Introduction

The ventral anterior (VA) nucleus of the thalamus is one of the major thalamic motor nuclei. It relays basal ganglia signals to the premotor cortex and holds a key position in planning and initiating movements [1, 2]. The motor symptoms of Parkinson's disease (PD) are thought to result from increased inhibitory outflow from the basal ganglia to the motor thalamus [3]. A reduced spontaneous firing rate of VA neurons has been reported in Parkinsonian patients [4] and the MPTP-induced cat model of PD [5]. Structural abnormalities of the VA have also been reported. A recent magnetic resonance imaging (MRI) study demonstrates a significant increase in the volumes of bilateral VA in PD patients [6]. However, little is known about the endogenous neuromodulation of the VA and its potential as a therapeutic target for treating the cardinal motor signs of PD.

Several lines of evidence suggest that, besides massive GABAergic afferents from the basal ganglia, the VA also receives neuromodulatory afferents, including histaminergic afferents from the tuberomammillary nucleus (TMN) of the posterior hypothalamus. Both histamine immunoreactive fibers and the mRNA expression of histamine receptors have been detected in the VA of humans and rats [7–9]. Moreover, histamine concentrations have been reported to be significantly increased in the basal ganglia of post-mortem brain samples from PD patients [10] and the 6-hydroxydopamine (6-OHDA) rat model of PD [11]. Notably, we have found that the rise of histamine levels in the basal ganglia may play a compensatory role in the amelioration rather than the deterioration of Parkinsonian motor deficits [11, 12]. Furthermore, deep brain stimulation (DBS) may increase histamine release in the subthalamic nucleus (STN) to alleviate the motor dysfunctions of PD [11]. However, the role and mechanisms of histamine and histaminergic innervation in the VA in normal motor control and Parkinsonian motor deficits remain unclear. In the present study, using functional magnetic resonance imaging (fMRI) on PD patients, as well as q-PCR, western blot, immunofluorescence staining, whole-cell patch clamp recording, and optogenetic and pharmacological manipulation combined with motor behavioral tests on the 6-OHDA rat model of PD, we explore the impact of histaminergic afferent inputs on VA neuronal activity and Parkinsonian motor deficits. The results showed that histamine and optogenetic activation of histaminergic afferents effectively increase VA neuronal excitability to improve motor performance in both normal and Parkinsonian rats. Postsynaptic H1 and H2 receptors co-mediate the excitatory effect of histamine on VA neurons *via* their downstream ionic channels, including the hyperpolarization-activated cyclic nucleotide-gated (HCN) channels, inward-rectifier K^+^ (Kir) channels, and Ca^2+^-activated K^+^ (SK) channels. These findings suggest that histamine receptors and the underlying ion channels may be potential targets for the clinical treatment of PD.

## Materials and Methods

### Human Participants

All participants with PD (*n* = 22, mean age = 63.81 ± 7.69 years, aged 50–75, gender M/F = 12/10) were recruited from the inpatient services at Nanjing Medical University Affiliated Brain Hospital and diagnosed with PD in accordance with the UK Parkinson’s Disease Brain Bank criteria. Matched healthy control participants (*n* = 21, mean age = 60.29 ± 7.72 years, aged 49–77, gender M/F = 9/12) were recruited at the same time. Participants who had obstructive sleep apnea syndrome, dementia, epilepsy, claustrophobia, or psychiatric disorders were excluded. Before the fMRI scan, all participants underwent comprehensive examinations during 12 h –15 h of the off-state. The clinical study was conducted in accordance with the principles of the Declaration of Helsinki and approved by the Medical Ethics Committee of Nanjing Brain Hospital Affiliated with Nanjing Medical University (2021-KY006). Written informed consent was given by all participants after a complete description of the study. Demographic and clinical data, including age, education, sex, disease duration, and clinical symptoms, were registered on the same day. Levodopa equivalent daily dose (LEDD) was calculated according to our previous research [13, 14].

### Experimental Animals

Adult male Sprague-Dawley rats and our established transgenic rat strain [15] expressing Cre recombinase in histidine decarboxylase (HDC, the histamine-synthesizing enzyme) neurons (220 g–250 g) were housed under a 12 h light/dark cycle, constant temperature, and humidity (21 °C–23 °C and 40%–60%) with free access to food and water. HDC-Cre rats were generated using CRISPR/Cas9 technology, as we reported previously [15]. Experimental procedures and animal care were in accordance with the Animal Ethics and Welfare Committee of Nanjing University (registration number IACUC-2005006), and the relevant regulations of the NIH Guide for the Care and Use of Laboratory Animals.

Unilateral 6-OHDA lesions (Sigma, St. Louis, USA) were made to establish a rat model of hemiparkinsonism as we reported previously [11]. Adult rats (220 g–250 g) were placed into a stereotaxic frame (1404, David Kopf Instruments, Tujunga, USA) after anesthesia (sodium pentobarbital, 40 mg/kg, *i*.*p*., Sigma). 6-OHDA (4 µL, 2.5 μg/μL, dissolved in saline containing 0.02% ascorbic acid; Sigma) was microinjected into the medial forebrain bundle (in mm: A − 4.4, L 1.8, and H 7.8) according to the rat brain atlas of Paxinos and Watson (2013) 30 min after treatment with desipramine hydrochloride (25 mg/kg, *i*.*p*.; Sigma) to protect noradrenergic neurons. Two weeks after surgery, the rats were injected with apomorphine (0.25 mg/kg, Sigma) and tested for rotational behavior. Only rats with significant turning behavior (> 25 turns in 5 min) contralateral to the lesion side were considered Parkinsonian rats and retained for further studies. At the end of the studies, the bilateral substantia nigra (SN) was immunostained for tyrosine hydroxylase (TH) to confirm ipsilesional dopaminergic depletion.

### Resting-State fMRI in Human Subjects

Resting-state fMRI was acquired in a 3.0 T MRI system (Siemens, Verio, Germany) equipped with a standard 8-channel head coil after anti-PD medications were discontinued for at least 12 h (i.e., during the off-stage) to decrease the medication’s influence on the images as we have reported previously [13, 16]. Subjects were instructed to lie flat, close their eyes, and remain awake in the process of scanning. To restrict head movement, sponge pads were placed on all subjects. T1 images were acquired using the 3D magnetization-prepared rapid gradient-echo (3D-MPRAGE) sequence with the following parameters: repetition time (TR) = 1900 ms, echo time (TE) = 2.48 ms, flip angle (FA) = 9°, matrix size = 256 × 256, field of view (FoV) = 250 mm × 250 mm, slice number = 176, slice thickness = 1 mm, and slice gap = 0 mm. The total scan time was 4 min 18 s. Functional images were obtained using an echo-planar imaging (EPI) sequence (TR = 2000 ms, TE = 25 ms, FA = 90, matrix size = 64 × 64, FOV = 240 mm × 240 mm, slice number = 33, slice thickness = 4 mm, and slice gap = 0 mm). The total scan time was 8 min 6 s.

As we reported previously [17], resting-state fMRI images were all preprocessed using SPM12 (https://www.fil.ion.ucl.ac.uk/spm/) and DPABI (http://rfmri.org/DPABI). The first 10 TRs were discarded to allow magnetization to reach a study state. Slice time correction and head motion correction were then processed. By using the EPI template in SPM12, the functional images were normalized to Montreal Neurological Institute (MNI) space, resampled to 3-mm voxels, and smoothed *via* a Gaussian kernel with a 6-mm full-width at half-maximum. Then, we applied linear detrending. Moreover, several confounding covariates, including the Friston-24 head motion parameters, white matter, and cerebrospinal fluid, were regressed from the BOLD time series for all voxels. We used Automated Anatomical Labelling Atlas 3 [18] to identify the VA nucleus as in previous studies [19, 20]. Finally, the amplitude of low-frequency fluctuation (ALFF) was calculated to measure brain activity. All the individual whole-brain ALFFs were standardized with the z-score. Then, the independent two-sample *t*-test was applied to the two groups under the mask of the VA nucleus, which was extracted from the atlas of the adult human brain. Finally, Generalized Ransom Forest correction was applied to the *t*-test result.

### Quantitative Reverse Transcription PCR (qRT‑PCR)

As we have reported previously [11], 5 independent groups of RNA pools, each from 3 animals, were used as biological replicates. VA tissue punches were collected from coronal brain slices from Sprague-Dawley rats (weighing 220 g–250 g) according to the atlas of Paxinos and Watson (2007) and pooled (3 animals in each pool). RNA was extracted using TRIzol reagent (Invitrogen, Carlsbad, USA) according to the manufacturer’s instructions. A 1 μg aliquot of total RNA was used for the first-strand cDNA synthesis according to the protocol of HiScript Reverse transcriptase (Vazyme, Nanjing, CN). Real-time PCR was then applied using qPCR SYBR Green Master Mix (Yeasen, Shanghai, CN) in 20 μL of reaction mixture containing 10 μL of qPCR SYBR Green Master Mix, 2 μL of cDNA, 0.4 μL of each primer (10 μmol/L), and 7.2 μL of distilled water. The reaction was carried out in a Roche light cycler 480 real-time PCR system using the following parameters: 95 °C for 30 s to activate the hot-start iTaq DNA polymerase, followed by 40 cycles at 95 °C for 10 s, 60 °C for 20 s, and 72 °C for 20 s. The PCR program was completed by a melting temperature analysis. The quantity of the target gene was expressed relative to the amount of the reference gene (*gapdh*) to obtain a normalized target expression value. For negative controls, cDNA was replaced with water. Primer sequences are as follows: mRNAs of GAPDH (forward: *TTCAACGGCACAGTCAAGG*, reverse: *CTCAGCACCAGCAT-CACC*), HRH1 (forward: *CACTTGAACCGAGAGCGGAA*, reverse: *CGTGGAGTTG-ATGTAGCCCA*), and HRH2 (forward: *ATTCGTTTACCGTGGGCTGA*, reverse: *GAGAGTTGTGGCT-TGCGAAC*).

### Western Blot

Western blot assays were performed as previously reported [11]. The VA tissue punches were homogenized in 500 μL cell lysate (50 mmol/L Tris, pH 7.4, 150 mmol/L NaCl, 1% Triton X-100, 1 mmol/L PMSF, 1% sodium deoxycholate, 0.1% SDS, 2.0 mmol/L EDTA, 10 μg/mL leupeptin). The protein concentration was assessed using a BCA protein assay kit (Thermo Fisher Scientific, Waltham, USA), followed by heating at 95 °C for 5 min. Caudate-putamen (Cpu) and lateral vestibular nucleus (LVN) tissue punches were also performed with the same treatments. The protein samples were separated using 10% SDS polyacrylamide gel, and the proteins in the gels were transferred onto nitrocellulose membranes, which were then placed in a 5% blocking agent for 1 h. The immunoblots were incubated overnight at 4 °C in the primary antibody including mouse anti-GAPDH (1:2000; Santa Cruz, Dallas, USA, Cat# SC-365062, RRID: AB_10847862), rabbit anti-HRH1 (1:200; Alomone Labs, Jerusalem, Israel, Cat# AHR001, RRID: AB_2039915), and goat anti-HRH2 (2 µg/mL; Everest Biotech, Upper Heyford, UK, Cat# EB06905, RRID: AB_2121375), and incubated at room temperature for 3 h in the secondary antibody including horseradish peroxidase (HRP)-conjugated anti-mouse antibody (1:5000; Thermo Fisher Scientific, Cat# 62-6520, RRID: AB_88369), HRP-conjugated anti-rabbit antibody (1:5000; Thermo Fisher Scientific, Cat# 62-6520, RRID: AB_88369), and HRP-conjugated anti-goat antibody (1:5000; Thermo Fisher Scientific, Cat# 31402, RRID: AB_228395). After washing with TBST (tris-buffered saline containing 0.2% Tween 20), the protein entity complexes were visualized by the Pierce ECL Western Blotting Substrate (Thermo Fisher Scientific) and exposed to Kodak medical X-ray film (Denville Scientific Inc., Holliston, USA).

### Immunofluorescence Staining

Immunostaining was applied as in our previous reports [15, 17, 21]. Rats of either sex were deeply anesthetized with sodium pentobarbital and transcardially perfused with 100 mL saline, followed closely by 250 mL–300 mL 4% paraformaldehyde or 4% *N*-(3-Dimethylaminopropyl)-*N*′-ethylcarbodiimide hydrochloride (EDAC; Sigma; for histamine immunostaining) in 0.1 mol/L phosphate buffer. Brains were immediately removed and postfixed in 4% paraformaldehyde at 4 °C overnight or 4% EDAC for 4 h followed by 4% paraformaldehyde at 4 °C overnight. Each brain was then dehydrated and cryoprotected by incubation in 20% sucrose, then 30% sucrose, for 24 h each. The frozen brain was cut into 25-μm-thick coronal sections containing the TMN, VA, and SN, which were stabilized on gelatin-coated slides by a freezing microtome (CM3050S, Leica, Germany). Sections were rinsed extensively in PBS containing 0.1% Triton X-100 (PBST) and then blocked in 10% normal bovine serum in PBST for 30 min.

The sections were incubated at 4 °C overnight with primary antibodies against tyrosine hydroxylase (rabbit anti-tyrosine hydroxylase antibody, 1:500; Abcam, Cambridge, UK, Cat# AB137869, RRID: AB_2801410), histidine decarboxylase (rabbit anti-histidine decarboxylase antibody, 1:200; Progen, Wayne, USA, Cat# 16045, RRID: AB_2773044), NeuN (rabbit anti-NeuN antibody, 1:500; Proteintech Group, St. Rosemont, USA, Cat# 26975-1-AP, RRID: AB_2880708), H1 receptor (rabbit anti-histamine H1 receptor antibody, 1:500; Alomone Labs, Cat# AHR-001, RRID: AB_2039915), and H2 receptor (goat anti-histamine H2 receptor antibody, 1:500; Everest Biotech, Cat# EB06905, RRID: AB_2121375). The sections were washed with PBS after each step and followed by the related secondary antibodies (1:2000; Invitrogen) conjugated to AlexaFluor 647, AlexaFluor 555, and AlexaFluor 488 for 2 h in the dark at room temperature. All high-resolution images were captured using a laser confocal microscope (LSM 980 with Airyscan 2; Zeiss, Oberkochen, Germany). The number of dopaminergic neurons in the SN was estimated by counting the number of neurons within 3D optical dissectors that were extensively spaced at random. Ten optical dissectors sized 100 μm × 100 μm × 50 μm were randomly examined, and the number of positive cells was quantified using the principle of the optical dissector. The density of cells was estimated using the following formula: Nv = Q/v (dis), where Q is the average number of cells counted per dissector, and v (dis) is the volume of the dissector: v (dis) = a [frame] × h, where a is the area of frame and h is dissector height. Data are presented as the number of cells per cubic millimeter.

### Whole-Cell Patch Clamp Recording

Brain slices containing the VA were cut at 300 μm on a vibratome (VT 1200S, Leica). Whole-cell patch-clamp recordings were performed on VA neurons in brain slices to assess the receptor and ionic mechanisms. The artificial cerebrospinal fluid used for whole-cell patch clamp recordings was as follows (in mmol/L): 124 NaCl, 2.5 KCl, 1.25 NaH_2_PO4, 1.3 MgSO_4_, 26 NaHCO_3_, 2 CaCl_2_, and 20 D-glucose, and was equilibrated with 95% O_2_ and 5% CO_2_. Whole-cell patch-clamp recordings were conducted using glass micropipettes (3 MΩ–5 MΩ) filled with an internal solution of (in mmol/L) 140 K-methyl sulfate, 7 KCl, 2 MgCl_2_, 10 HEPES, 0.1 EDTA, 4 Na_2_-ATP, and 0.4 GTP-Tris, adjusted to pH 7.25 with 1 mol/L KOH. The VA neurons were visualized under an Olympus BX51W1 microscope (Olympus, Tokyo, Japan) equipped with infrared differential interference contrast. An Axopatch-700B amplifier (Axon Instruments, Foster City, USA) was used for whole-cell patch-clamp recordings. The signals were fed into a computer through a Digidata-1550 interface (Axon Instruments) for data capture and analysis (pClamp 10.0, Axon Instruments).

The dosages of drugs we applied were in line with our previous reports [13, 22, 23]. Before bath application, the whole-cell current of a neuron was recorded for at least 10 min to assure stability. Then histamine (30 μmol/L–300 μmol/L, Tocris, Bristol, UK) was administered by bath application (1 min). At least 20 min was allowed for cells to recover after drug application to avoid desensitization. Whether the effect of histamine was postsynaptic was assessed by combined application of tetrodotoxin (TTX; 0.3 μmol/L, Alomone Labs), the selective AMPA receptor antagonist NBQX (10 μmol/L, Tocris), the selective NMDA receptor antagonist D-AP5 (10 μmol/L, Tocris), and the selective GABA_A_ receptor antagonist SR 95531 (10 μmol/L, Tocris). Selective agonists for the histamine H1 receptor (2-PyEA, 300 μmol/L, Tocris) and H2 receptor (dimaprit, 300 μmol/L, Tocris), as well as selective antagonists for the H1 receptor (mepyramine, 3 μmol/L, Tocris) and the H2 receptor (ranitidine, 3 μmol/L, Tocris) were applied to examine the underlying postsynaptic receptor mechanism. To characterize the histamine-induced whole-cell current, in voltage-clamp recording, current-voltage plots (*I*–*V* curves) were constructed before and during activation of histamine H1/H2 receptors with 2-PyEA/dimaprit using a slow ramp command (*dV/dt* = − 10 mV/s, ranging from − 60 mV to − 120 mV) to allow the attainment of steady-state conditions. To verify the coupled channels underlying H1 and H2 receptors, ZD7288 (50 μmol/L, Tocris), Tertiapin-Q (100 nmol/L, Tocris), and apamin (100 nmol/L, Tocris) were applied. Moreover, a direct 50-pA hyperpolarizing current was injected to determine the impact of histamine and the ion channels coupled to histamine receptors on the rebound firing of VA neurons.

### Stereotaxic Implantation and Microinjection

Male rats (220 g–250 g) were anesthetized with sodium pentobarbital (40 mg/kg) intraperitoneally and then mounted on a stereotaxic frame (1404, David Kopf Instruments) for stereotactic brain surgery under aseptic conditions. A heating pad was used to maintain rectal temperature at 36 °C–38 °C. Briefly, two stainless-steel guide tubes (length 11 mm, o.d. 0.8 mm, i.d. 0.5 mm) for the microinjection cannulae were implanted into the bilateral VAs of each rat. The lower ends of the guide tubes were positioned 2.0 mm above the VA (in mm: A − 2.04, L 1.65, and H 5.8). After surgery, animals were caged individually and allowed to recover for at least 3 days.

For microinjection into the VAs, two injection cannulae (length 13 mm, o.d. 0.5 mm, i.d. 0.3 mm) were inserted to protrude 2 mm beyond the tip of the guide tube. The lower ends of the injection cannulae were just above the bilateral VAs to minimize lesioning of the nuclei. Saline (0.9% NaCl), selective antagonists for the H1 receptor (mepyramine, 3.5 μg, Tocris), and H2 receptor (ranitidine, 4 μg, Tocris) were microinjected with Hamilton syringes (0.5 µL per side, lasting 2 min). Based on our previous studies [11, 17, 24, 25], the effective diffusion ranges of drugs were determined by extracellular electrophysiological recording, and the injection drug doses in this study were tested to ensure that the diffusion areas of drugs were limited to the VA. Data from rats in which implantation sites were histologically identified to deviate from the target brain regions were excluded from further analysis.

### Optogenetic Manipulation

For virus injections, VA (in mm: A − 2.04, L 1.65, and H 5.8) or TMN (A − 4.5, L 1.2, and H 9.6) was infused bilaterally with 0.4 µL of rAAV2/9-hSyn-hChR2-mCherry (Taitool, Shanghai, China) or rAAV2/9-hSyn-DIO-hChR2-EGFP (Taitool) through 33-gauge needles.

As we reported previously [15, 17], the single end of a 2 × 1 fiber splitter (Newdoon, Hangzhou, CN) was connected to a rotating commutator (Doric, Quebec, Canada), which was then attached *via* a fiber to a laser (Newdoon). Light output was measured with an optical power meter and adjusted to 7 mW of 473-nm light. For photostimulation of VA neurons or TMN-VA histaminergic afferent terminals during the behavioral tests, blue light was applied at a pulse width of 10 ms and a frequency of 10 Hz.

### Behavioral Tests

#### Open-Field Test

As we have reported previously [11, 15, 24], spontaneous locomotor activity was evaluated by locating individual rats for 10 min in an open-field arena (50 cm × 50 cm × 50 cm) with a video camera positioned at the top of the arena. Rats were placed in the center of the arena at the beginning of the open-field test. Total travel distance and instantaneous velocity were quantified to estimate locomotion. The percentage of time spent in the central zone (25 cm × 25 cm) was used to measure anxiety.

#### Rotarod Test

As in our previous reports [11, 15, 24], an accelerating rotarod test was applied to estimate motor coordination and balance. For this test, rats were subjected to 3 trials, with 5 min inter-trial intervals to eliminate fatigue and stress. The average latency to fall from the rod in a session (3 repeat trials) or cling to and rotating with the rod was recorded to assess rotarod performance. Rats were adapted to the treadmill (Model 47650, Ugo Basile, Varese, Italy) before the accelerating rotarod test and were required to keep walking and avoid dropping from the rod for 15 s. When testing, the rotation speed was initially set at 5 r/min for 15 s, and then increased to 40 r/min for 300 s.

#### Turning Behavior Test

As we have reported previously [11], the 6-OHDA-lesioned rats were given an intraperitoneal injection of apomorphine (0.25 mg/kg, *i*.*p*.) 3 weeks after surgery. The number of rotations contralateral to the lesion was counted 30 min after apomorphine injection.

#### Adhesive-Removal Test

As we have reported previously [11], the adhesive-removal test was applied to assess motor initiation and execution. Two training trials were conducted by placing two adhesive tapes (8 mm × 6 mm) on the plantar surface of both forelimbs simultaneously. The rat may bring one forelimb onto the other forelimb and use its mouth to remove the adhesives. We trained the animals for 5 days (1 trial per animal per day; each trial took a maximum of 3 min). On day 6, the time for PD rats to remove the tape was recorded. If a rat failed to remove either or both stickers within 60 s, a score of 60 s was recorded.

#### Wire-Hanging Test

As has been reported previously [26, 27], the wire-hanging test was applied to assess grip strength and coordination. A metallic wire (2 mm in diameter, 60 cm long) was stretched horizontally between 2 vertical stands, 50 cm above the ground, the rat was placed midway and then left for a maximum of 5 min. A foam pad was situated beneath the rat to prevent injury due to falls. Each rat was experimenter-rated according to the following scores: 0, falls off; 1, hangs onto string by two forepaws; 2, as for 1, but attempts to climb to the string; 3, hangs onto the string by two forepaws plus one or both hind paws; 4, hangs onto the string by forepaws with tail wrapped around string; 5, escapes.

### Statistical Analysis

All data are expressed as the mean ± SEM and were analyzed using SPSS 17.0 software. Student’s *t*-test or one-way analysis of variance (ANOVA) was applied to determine the statistical significance of differences. *P* values < 0.05 were considered to be significantly different.

## Results

### VA Neuronal Activity is Decreased in PD Patients

**Fig. 1:**
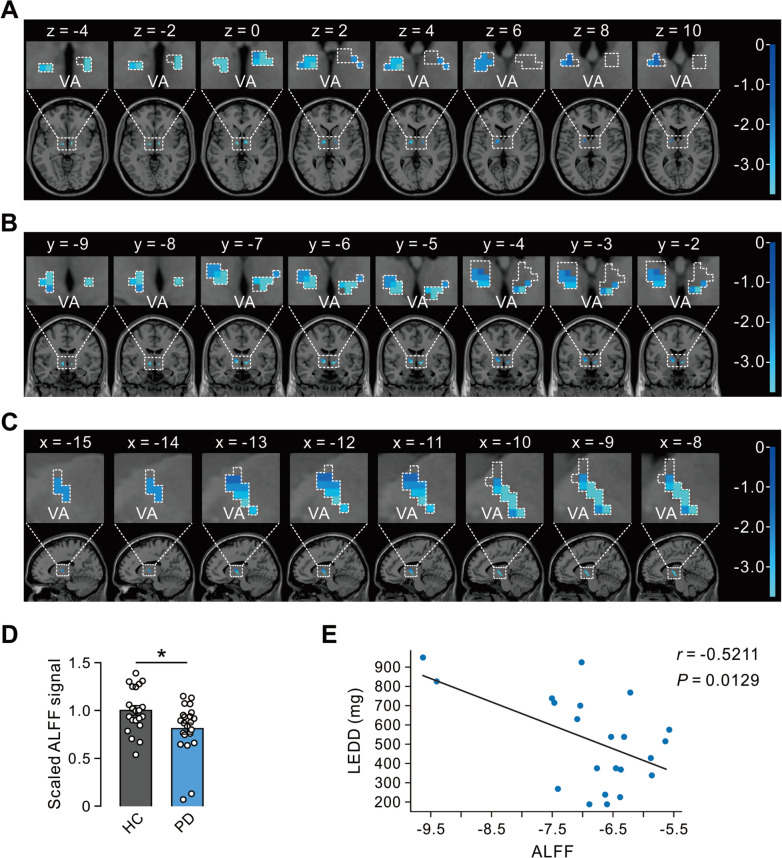
Thalamic VA activity is decreased in PD patients. **A**–**C** Resting-state fMRI images from PD patients along the x, y-, and z-directions to show the ALFF. The area enclosed by a dashed line indicates the range of the VA identified by Automated Anatomical Labelling Atlas 3. Blue, areas of the VA with decreased ALFF in PD patients; color bar, the size of the T value. **D** The ALFF in the VA in the healthy group (HC, *n *= 21) and the PD group (*n *= 22) is significantly different (*P *= 0.0178) (the ALFF data is scaled with the mean value of the healthy group). **E** A negative correlation between L-dopa equivalent daily dose (LEDD) and VA ALFF value in PD patients (*n *= 22, *P *= 0.0129). *P < 0.05, unpaired two-tailed Student’s *t*-test (**D**), Pearson correlation coefficient test, and simple linear regression (**E**).

We collected resting-state fMRI data from patients with PD to evaluate the pathological changes in the activity of the VA [28]. We determined the ALFF of the resting-state fMRI signal, which reflects the intensity of regional spontaneous brain activity [29]. Compared with healthy control subjects, PD patients showed a decreased ALFF in the VA (Fig. 1A–D). This result indicates a lower baseline brain activity of the VA in PD patients, which is in line with the previous report of neuronal recordings during microelectrode-guided functional stereotactic neurosurgery in PD patients [4]. Moreover, the ALFF in the VA had a significant negative correlation with the LEDD (Fig. 1E).

### Activation of VA Neurons Alleviates Parkinsonian Motor Deficits

**Fig. 2:**
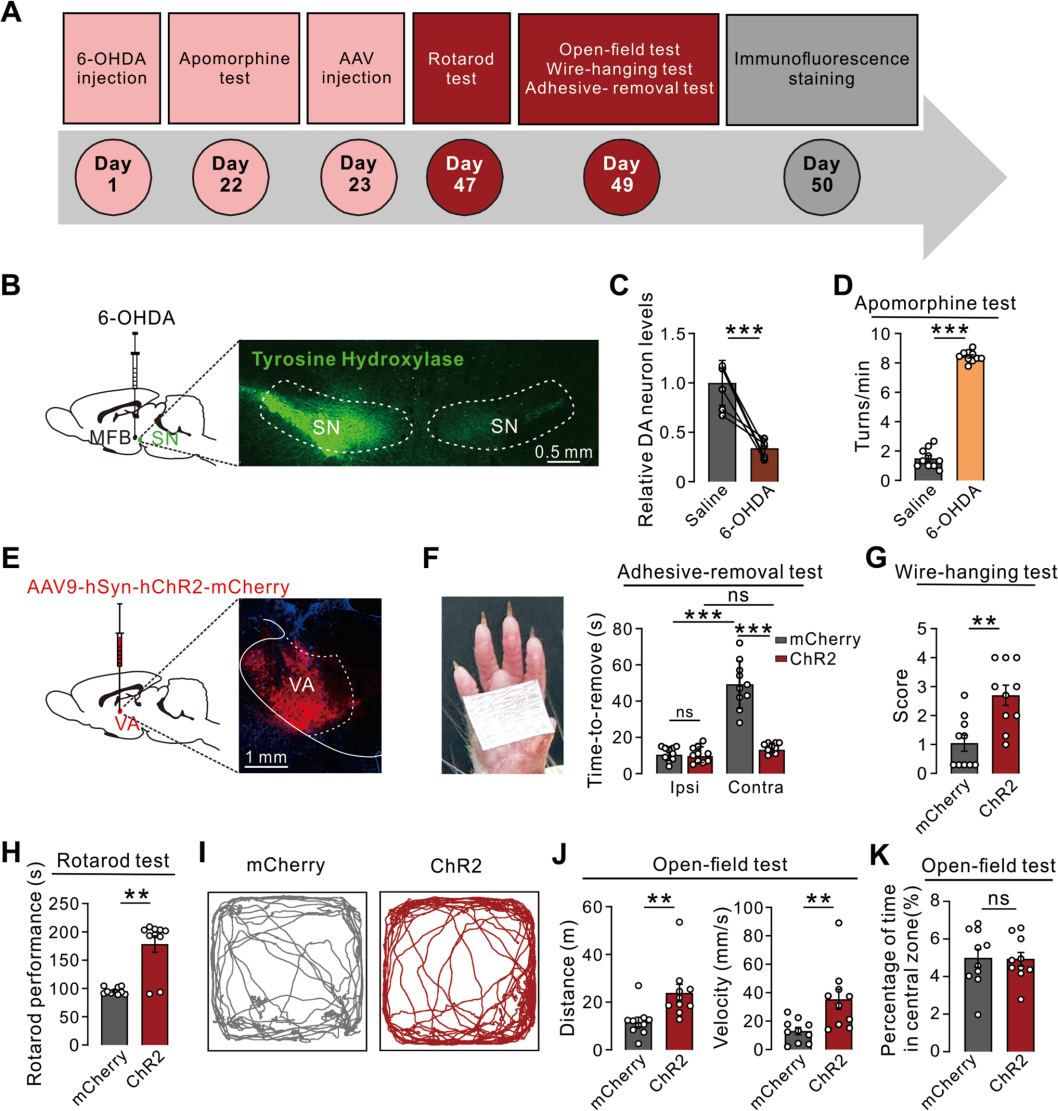
Optoactivation of VA ameliorates Parkinsonian motor deficits. **A** Timeline of the behavioral tests. **B**, **C** The population of TH-positive dopaminergic neurons in the ipsilesional SNc is significantly reduced in 6-OHDA-lesioned rats (*n *= 6 rats, *P *< 0.001). **D** 6-OHDA-lesioned rats exhibit pronounced contralateral circling upon intraperitoneal administration of apomorphine (*n *= 10 rats, *P *< 0.001). **E** AAV vector containing ChR2-mCherry is locally microinjected into the VA. **F** Optogenetic activation of VA neurons significantly shortens the prolonged contralesional adhesive-removal time in PD rats (*n *= 10 rats, *P *< 0.001), and restores the contralesional adhesive-removal time to the control level of the ipsilateral side (*n *= 10 rats, *P *= 0.0582). **G** Optogenetic activation of VA neurons increases the performance of PD rats in the wire-hanging test (*n *= 10 rats, *P *= 0.0027). **H** Optogenetic activation of VA neurons largely extends the time for 6-OHDA-lesioned rats to fall off the rotarod (*n *= 10 rats, *P *= 0.0042). **I**–**K** Optogenetic activation of VA neurons increases the total locomotor distance (*n *= 10, *P *= 0.0011) and velocity (*n *= 10, *P *= 0.0039) of PD rats in an open field (**J**), but does not change the percentage of time spent in the central zone (*n *= 10, *P *= 0.9705) (**K**). Scale bars, 0.5 mm (**B**), 1 mm (**E**). Data are shown as the mean ± SEM. ns indicates no significant difference. *P < 0.05, **P < 0.01, ***P < 0.001, paired two-tailed Student’s *t-*test (**C**) or unpaired two-tailed Student’s *t-*test (**D**, **F**, **G**, **H**, **J**, and **K**).

We further assessed whether activating VA neurons could alleviate Parkinsonian motor deficits (Fig. 2A). A rat model of PD was generated by unilateral microinjection of 6-OHDA into the medial forebrain bundle. Three weeks after 6-OHDA microinjection, the population of TH-immunopositive dopaminergic neurons in the SNc region ipsilateral to the injection site was significantly reduced (Fig. 2B, C). Moreover, 6-OHDA-lesioned rats exhibited pronounced contralateral circling behavior upon intraperitoneal administration of apomorphine (Fig. 2D), confirming the ipsilateral impairment of nigrostriatal dopaminergic signaling 21 days after 6-OHDA infusion. We next determined the effect of optogenetic activation of the VA on Parkinsonian motor dysfunction. An AAV vector containing ChR2-mCherry or mCherry alone under the control of the hSyn promotor was locally microinjected into the VA (Fig. 2E). In the adhesive-removal test, 6-OHDA-lesioned rats took longer to remove a rectangular adhesive strip from the contralesional forelimb, compared to the ipsilesional forelimb (Fig. 2F). Blue light stimulation of VA neurons (10 ms pulse width at 10 Hz) significantly reduced the prolonged contralesional adhesive-removal time in PD rats (Fig. 2F). Notably, the rescued contralesional adhesive-removal time showed no significant difference from the control level of the ipsilateral side (Fig. 2F). These results indicate that an increase in VA neuronal activity may improve the slowness of motor initiation and execution in PD rats. Moreover, optoactivation of VA neurons remarkably increased the motor performance of PD rats in the wire-hanging test (Fig. 2G), suggesting an amelioration of muscle weakness, a frequent PD symptom [30, 31]. Furthermore, optoactivation of VA neurons largely extended the time to fall off the rotarod (Fig. 2H), and substantially increased the total movement distance and velocity of PD rats in an open field (Fig. 2I, J), suggesting that VA activation may also improve spontaneous locomotor activity and motor coordination in PD rats. Since individuals with PD also experience non-motor symptoms (e.g., anxiety) [32, 33], we further estimated the anxiety-related behaviors of PD rats in the open field. Optoactivation of VA neurons did not affect the percentage of time PD rats spent in the center areas of the open field (Fig. 2K), indicating that VA neurons may not be involved in the regulation of anxiety in PD. Therefore, optogenetic activation of the VA in rats alleviates Parkinsonian motor symptoms, including the slowness of motor initiation and execution and muscle weakness, as well as motor incoordination and imbalance.

### Histamine Directly Excites VA Neurons via Postsynaptic H1 and H2 Receptors

**Fig. 3:**
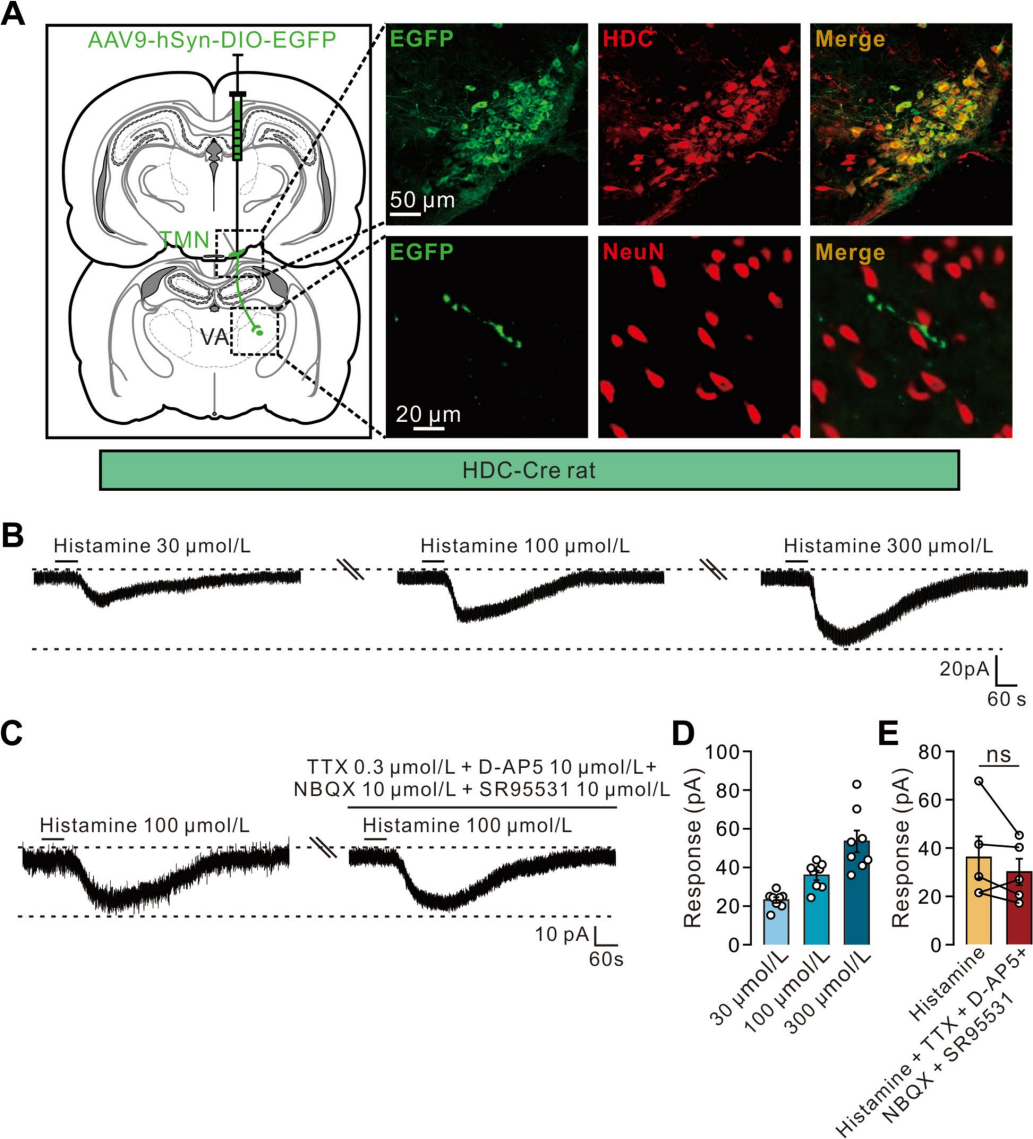
Hypothalamic TMN neurons directly project to the VA and histamine postsynaptically excites VA neurons. **A** Delivery of a Cre-dependent monosynaptic anterograde tracer AAV2/9-hSyn-DIO-EGFP into the hypothalamic TMN of HDC-Cre rats shows that EGFP-positive neurons are colocalized with HDC-labeled TMN neurons, and EGFP-positive fibers are observed in the VA around NeuN-labeled VA neurons. **B** Histamine (30, 100, and 300 μmol/L) concentration-dependently evokes an inward current on a VA neuron. **C** Co-application of TTX, NBQX, D-AP5, and SR95531 does not block the histamine-elicited inward currents in a VA neuron. **D** Mean inward currents evoked by 30, 100, and 300 μmol/L histamine on VA neurons (*n *= 8 neurons from 6 rats). **E** Group data of the VA neurons in (**C**) (*n *= 5 neurons from 5 rats, *P *= 0.264). Scale bars, 50 μm (TMN), 20 μm (VA) (**A**), 20 pA, 60 s (**B**), 10 pA, 60 s (**C**). Data are presented as the mean ± SEM. ns indicates no significant difference, paired two-tailed Student’s *t*-test (**E**).

To investigate whether the histaminergic hypothalamic neurons project directly to VA neurons, we delivered the Cre-dependent monosynaptic anterograde tracer AAV2/9-hSyn-DIO-EGFP into the hypothalamic TMN (the sole origin of the central histaminergic system [34]) of a transgenic rat strain expressing Cre recombinase in HDC (the histamine-synthesizing enzyme) neurons that we constructed [15]. As shown in Fig. 3A, The distribution of EGFP-positive neurons was restricted to the hypothalamic TMN and colocalized with the HDC-labeled neurons, indicating that the expression of Cre is highly specific to central histaminergic neurons. We further observed that EGFP-positive fibers possessed prominent varicosities and passed around NeuN-labeled VA neurons (Fig. 3A), suggesting a direct projection of TMN histaminergic neurons to the VA and a possible modulation of VA neuronal activity by histamine released from the varicosities of histaminergic hypothalamic afferents.

Next, we made whole-cell patch clamp recordings from thalamic slices to assess the effect of histamine on VA neurons. Bath application of 30, 100, and 300 μmol/L histamine evoked significant inward currents of 23.1, 35.9, and 53.4 pA, respectively, in a concentration-dependent manner (Fig. 3B, D). Moreover, co-application of TTX, NBQX, D-AP5, and SR95531 did not block the histamine-elicited inward currents in the VA neurons (Fig. 3C, E), indicating a direct postsynaptic effect of histamine.

**Fig. 4:**
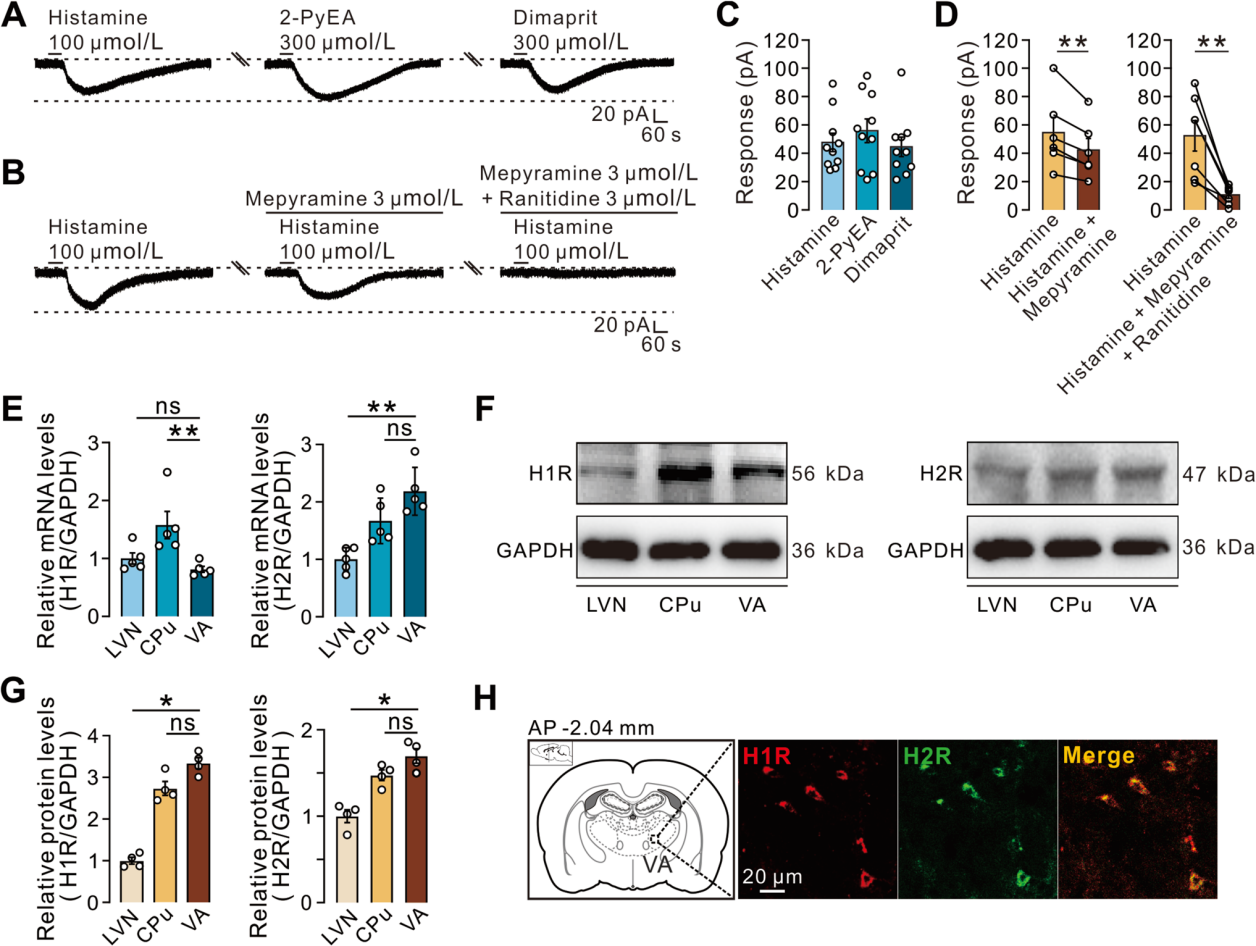
Histamine excites VA neurons *via* postsynaptic H1 and H2 receptors. **A** 2-PyEA (300 μmol/L) or dimaprit (300 μmol/L) mimics the whole-cell inward current induced by histamine (100 μmol/L). **B** The histamine-elicited inward current is partly blocked by mepyramine (3 μmol/L) and abolished by the combined administration of mepyramine (3 μmol/L) and ranitidine (3 μmol/L). **C** Mean inward currents evoked by 100 μmol/L histamine, 300 μmol/L 2-PyEA, and 300 μmol/L dimaprit on the tested VA neurons (*n *= 10 neurons from 8 rats). **D** Group data of the tested VA neurons (mepyramine: *n *= 6 neurons from 5 rats, *P *= 0.0053; mepyramine and ranitidine: *n *= 7 neurons from 6 rats, *P *= 0.0031). **E** The levels of histamine H1 mRNA (*n *= 5 samples from 15 rats, VLN-VA: *P *= 0.0952; CPu-VA: *P *= 0.0079) and H2 receptors (*n *= 5 samples from 15 rats, LVN-VA: *P *= 0.0079; CPu-VA, *P *= 0.1508) in the VA by q-PCR. **F**, **G** The levels of H1 protein (*n *= 4 samples from 4 rats, *P *= 0.0286) and H2 receptors (*n *= 4 samples from 4 rats, *P *= 0.0286) in the VA by western blot. **H** Histamine H1 and H2 receptors co-expressed in the same VA neurons. Scale bars, 20 pA, 60 s (**A**, **B**), 20 μm (**H**). Data are shown as the mean ± SEM. ns indicates no significant difference. *P < 0.05, **P < 0.01, paired two-tailed Student’s *t*-test (**D**) or one-way ANOVA followed by Dunnett’s *post hoc* test (**E**, **G**).

Four distinct G protein-coupled receptors, H1–H4, are known to mediate the effects of histamine (Haas and Panula [34]). Among them, H1 and H2 are major subtypes located postsynaptically in the brain [35]. We thus determined whether the histamine-induced excitation of VA neurons is mediated *via* H1 and H2 receptors. As shown in Fig. 4A, C, both 2-PyEA (300 μmol/L, a selective agonist for the H1 receptor) and dimaprit (300 μmol/L, a selective agonist for the H2 receptor) were able to mimic the whole-cell inward current induced by histamine (100 μmol/L). Furthermore, this current was partly blocked by bath application of mepyramine (3 μmol/L, a selective antagonist for the H1 receptor), and abolished by combined administration of mepyramine and ranitidine (3 μmol/L, a selective antagonist for the H2 receptor) (Fig. 4B, D). In line with the results of patch-clamp recording, qPCR (Fig. 4E) and western blots (Fig. 4F, G) showed that both the mRNAs and proteins of the histamine H1 and H2 receptors are expressed in the VA. Their expression levels were comparable to those in the LVN and CPu (Fig. 4E–G), regions with abundant distribution of H1 and H2 receptors (Lin et al. [9]). Moreover, immunofluorescence staining showed the co-expression of H1 and H2 receptors in the same VA neurons (Fig. 4H). All these results suggest that histamine excites VA neurons *via* postsynaptic H1 and H2 receptors.

### HCN/Kir Channels Coupled to H1 Receptors and HCN/SK Channels Coupled to H2 Receptors Mediate the Excitatory Effect of Histamine on VA Neurons

**Fig. 5:**
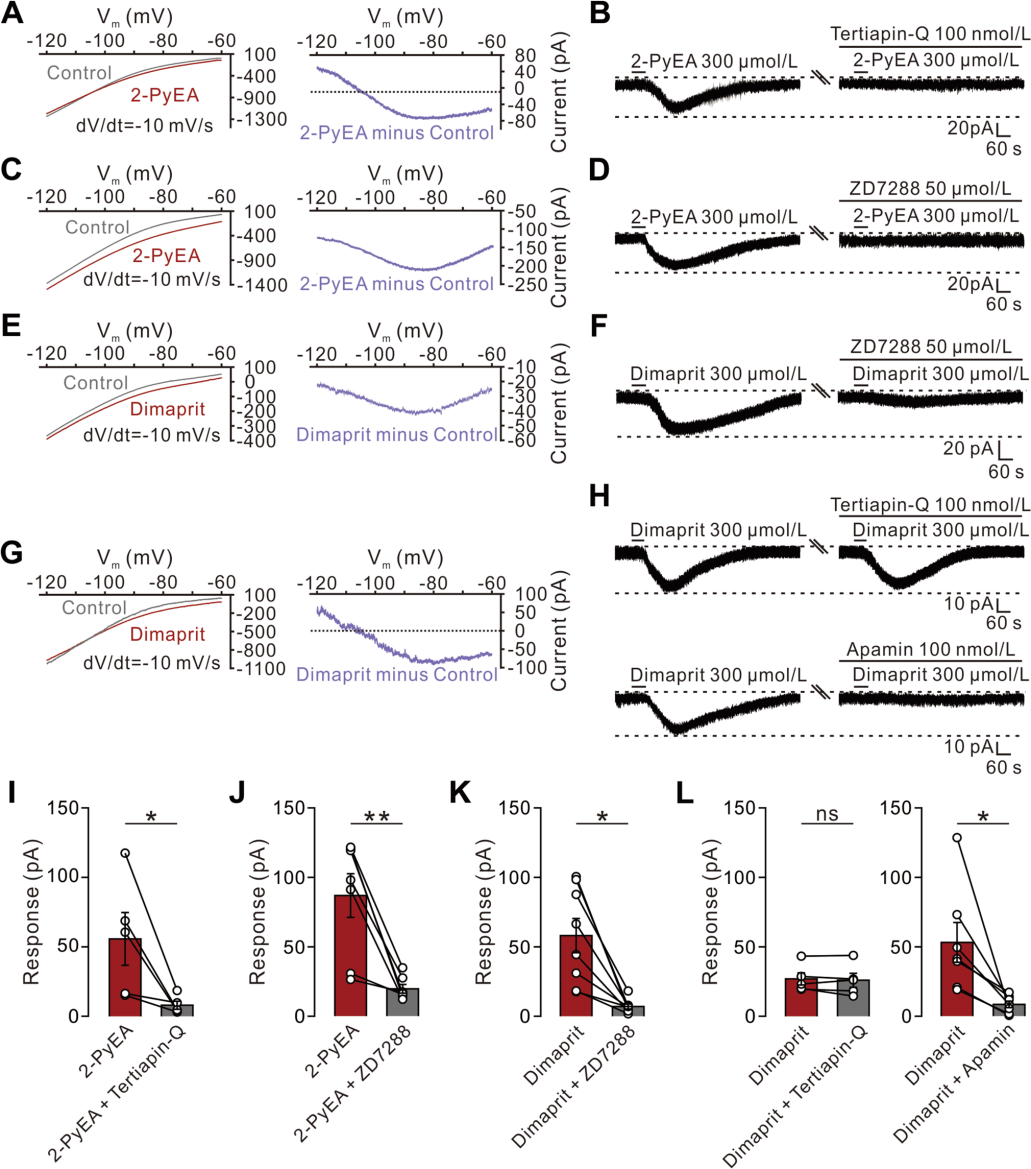
HCN/Kir channels coupled to H1 receptors and HCN/SK channels coupled to H2 receptors co-mediate the excitation of VA neurons by histamine. **A** 2-PyEA-induced change of *I–V* curves in VA neurons (*n *= 5) responding to a slow-ramp command indicates an involvement of Kir channels. **B** Tertiapin-Q (100 nmol/L) blocks the 2-PyEA-elicited inward current on the tested VA neurons in (**A**). **C** 2-PyEA-induced changes of *I–V* curves in VA neurons (*n *= 7) responding to a slow-ramp command indicate an involvement of HCN channels. **D** ZD7288 (50 μmol/L) blocks the 2-PyEA-elicited inward current in the tested VA neurons in (**C**). **E** Dimaprit-induced changes of *I–V* curves in VA neurons (*n *= 8) responding to a slow-ramp command indicate an involvement of HCN channels. **F** ZD7288 (50 μmol/L) blocks the dimaprit-elicited inward current in the tested VA neurons in (**E**). **G** Dimaprit-induced changes of *I–V* curves in VA neurons (*n *= 7) responding to a slow-ramp command indicate an involvement of K^+^ channels. **H** Apamin (100 nmol/L) rather than tertiapin-Q (100 nmol/L) blocks the dimaprit-elicited inward current in the tested VA neurons in (**G**). **I–L** Group data of the tested VA neurons (I: *n *= 5 neurons from 5 rats, *P *= 0.0218; J: *n *= 7 neurons from 6 rats,* P *= 0.0033; K: *n *= 8 neurons from 6 rats, *P *= 0.0209; **L** left: *n *= 5 neurons, from 5 rats, *P *= 0.524; **L** right: *n *= 7 neurons from 7 rats, *P *= 0.0204). Scale bars, 20 pA, 60 s (**B**, **D**, **F**); 10 pA, 60 s (**H**). Data are presented as the mean ± SEM. ns, no significant difference. *P < 0.05, **P < 0.01, paired two-tailed Student’s *t-*test (**I–L**).

To evaluate the ionic basis underlying the excitation of VA neurons induced by histamine, we used 2-PyEA and dimaprit to separate the current mediated by H1 or H2 receptors, respectively. We applied a slow-ramp command [11, 17] to assess the dynamic features of current induced by the activation of H1 receptors. Subtracting the control from the current recorded during the 2-PyEA application yielded a different current representing the 2-PyEA-induced current. Among 41.7% (5/12) of tested neurons, the difference current had a reversal potential of -105 mV that was near the calculated E_K_ (Fig. 5A, left) and was strongly outwardly rectifying (Fig. 5A, right), indicating that H1 receptor activation may close the inward rectifier K^+^ (Kir) channels to excite these VA neurons. To confirm this result, we further applied tertiapin-Q (100 nmol/L, a selective blocker of Kir channels) and found that the 2-PyEA-elicited inward current was nearly totally blocked on 41.7% (5/12) recorded VA neurons (Fig. 5B, I), suggesting that closure of Kir channels accounts for the excitation of these VA neurons induced by H1 receptor activation. In the rest of the recorded VA neurons (58.3%, 7/12), the difference current exhibited a significant feature of hyperpolarization activation (Fig. 5C), the characteristics of HCN channels [36]. Blockade of HCN channels with their selective antagonist ZD7288 (50 μmol/L) abolished the 2-PyEA-induced inward current (Fig. 5D, J). These results together suggest that the closure of Kir channels and/or the opening of HCN channels underlie the excitation of VA neurons induced by the activation of histamine H1 receptors.

The ionic mechanism underlying the inward current induced by the activation of H2 receptors was determined using dimaprit. Two types of *I-V* curves were found after applying dimaprit (Fig. 5E, G), indicating that a dual ionic mechanism is involved in the H2 receptor-mediated excitation of VA neurons. Similar to the characteristic of the 2-PyEA-induced current, the difference current representing the dimaprit-induced current in 15 recorded VA neurons showed either a significant HCN channel activation (i.e., hyperpolarization activation, 53.3%, 8/15) or a reversal potential near the calculated E_K_ with an outwardly rectifying property (46.7%, 7/15). In line with the results of the slow ramp test, in the 53.3% VA neurons tested, the dimaprit-induced current was nearly totally blocked after the bath application of ZD7288 (Fig. 5F, K). In addition, to clarify the identity of the dimaprit-induced K^+^ current component, we assessed the impact of blockade of Kir channels in the remaining 46.7% of tested neurons. As shown in Fig. 5H and L, the bath application of tertiapin-Q did not block the inward current on VA neurons elicited by dimaprit. Considering that the SK channel is also a known downstream ionic component coupled to H2 receptors in the brain, exhibiting the characteristics of an inward rectifier [37, 38], we applied apamin (100 nmol/L), a selective blocker of SK channels, and found that the dimaprit-induced excitation of VA neurons was eliminated (Fig. 5H, L). Taken together, these results suggest that HCN/Kir channels coupled to H1 receptors and HCN/SK channels coupled to H2 receptors co-mediate the excitatory effect of histamine on VA neurons.

### Histamine Promotes Rebound Firing of VA Neurons via the Activation of HCN Channels

**Fig. 6:**
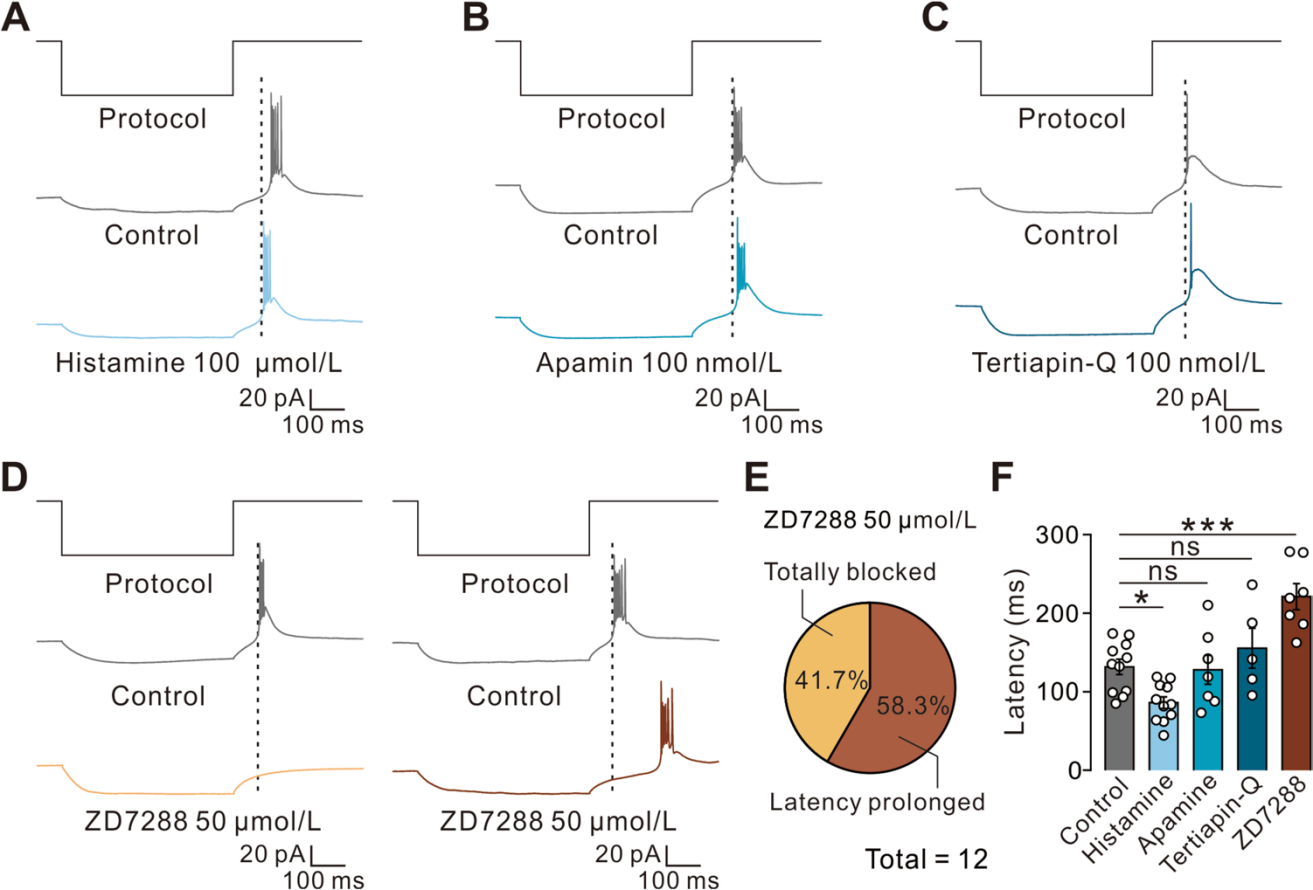
Histamine promotes rebound firing in VA neurons *via* the activation of HCN channels. **A** Histamine (100 μmol/L) application significantly reduces the latency of rebound firing of VA neurons induced by a direct hyperpolarizing current injection (50 pA) (F, *n *= 11 neurons from 8 rats, *P *= 0.0344). **B**, **C** Neither apamin (100 nmol/L) (F, *n *= 7 neurons from 5 rats, *P *= 0.999) nor tertiapin-Q (100 nmol/L) (F, *n *= 5 neurons from 5 rats, *P *= 0.629) affect the onset of post-inhibitory rebound firing in VA neurons. **D** ZD7288 (50 μmol/L) remarkably delays (*n *= 7 neurons from 5 rats, *P *= 0.0001) and even inhibits the rebound firing of VA neurons (*n *= 5 neurons from 4 rats). **E** The ratio of prolonging and inhibitory effects of ZD7288 on VA neurons. **F** Group data of the tested VA neurons. Data are shown as the mean ± SEM. ns, no significant difference. Scale bars, 20 pA, 100 ms (**A–D**). *P < 0.05, ***P < 0.001, one-way ANOVA followed by Dunnett’s *post hoc* test (**F**).

Since post-inhibitory rebound excitation is an intrinsic property of VA neurons that is implicated in normal motor behaviors and Parkinsonian motor dysfunctions [39, 40], we further assessed the impact of histamine on this rebound firing. Application of histamine (100 μmol/L) significantly reduced the latency of rebound firing of VA neurons induced by direct hyperpolarizing current injection (50 pA) (Fig. 6A). Next, we determined the contribution of ion channels coupled to histamine H1 and H2 receptors to the post-inhibitory rebound excitation in VA neurons. The results showed that neither blockade of SK channels *via* apamin (100 nmol/L; Fig. 6B) nor blockade of kir channels by tertiapin-Q (100 nmol/L; Fig. 6C) affected the onset of post-inhibitory rebound firing. In contrast, this rebound firing was remarkably delayed (7/12, 58.33%) and even totally suppressed (5/12, 41.67%) by ZD7288 (50 μmol/L) (Fig. 6D, E), suggesting that histamine promotes the rebound firing of VA neurons *via* the activation of HCN channels.

### Endogenous Histamine in the VA Improves the Motor Performance of Normal Rats

**Fig. 7:**
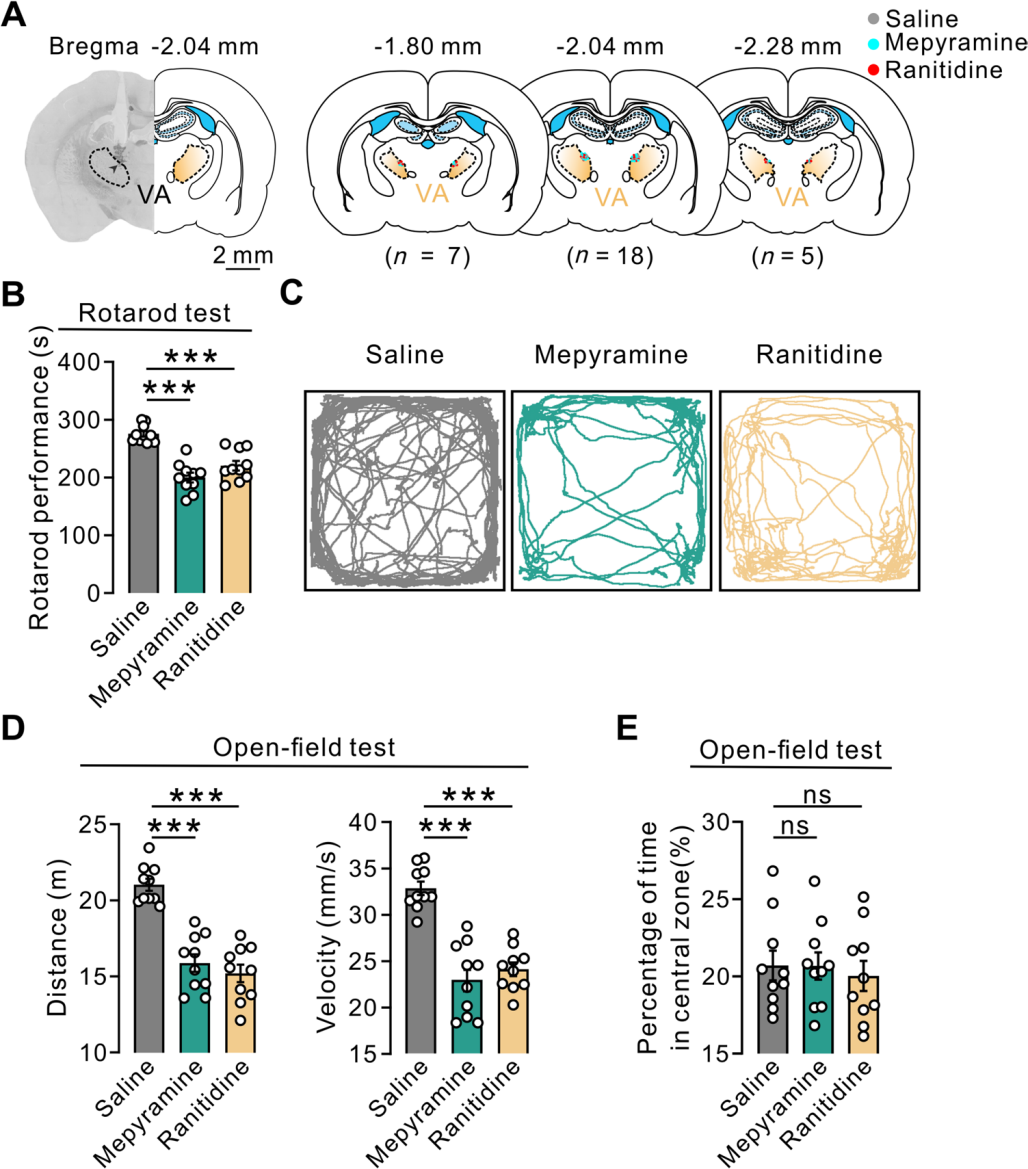
Blockade of histamine H1 and H2 receptors reduces motor performance of normal rats. **A** Histological identification of microinjection sites in the VA (*n *= 30 rats). **B** Intra-VA microinjection of mepyramine (*n *= 10 rats, *P *< 0.0001) or ranitidine (*n *= 10 rats, *P *< 0.0001) remarkably shortens the endurance time of PD rats on the rotating rotarod. **C–E** Intra-VA microinjection of mepyramine (*n *= 10 rats, *P *< 0.0001) or ranitidine (*n *= 10 rats, *P *< 0.0001) markedly decreases the locomotor distance and velocity of 6-OHDA-induced PD rats in an open field (**D**), while it does not change the percentage of time spent in the central zone (mepyramine: *n *= 10 rats, *P *= 0.7817; ranitidine: *n *= 10 rats, *P *= 0.5787) (**E**). Scale bar, 2 mm (**A**). Data are shown as the mean ± SEM. ***P < 0.001, one-way ANOVA followed by Dunnett’s *post hoc* test (**B**, **D**, **E**).

Next, we blocked histamine H1 and H2 receptors separately in the VA to determine the role of the endogenous histaminergic system in motor control (Fig. 7). The microinjection sites of drugs in the VA were histologically verified and reconstructed (Fig. 7A). Intra-VA microinjection of either mepyramine or ranitidine, to separately antagonize H1 or H2 receptors, remarkably shortened the endurance time of rats on the rotating rod (Fig. 7B). In the open-field test, the distance traveled and movement velocity were both significantly reduced after microinjection of mepyramine or ranitidine (Fig. 7C, D). Moreover, similar to the results of opto-manipulation of VA neurons, intra-VA microinjection of mepyramine or ranitidine did not alter the percentage of time spent in the central zone of the open field (Fig. 7E). These behavioral results indicate that endogenous histamine in the VA promotes motor performance rather than anxiety-related behaviors in normal rats.

### Optogenetic Activation of Histaminergic Afferents in the VA Ameliorates Parkinsonian Motor Dysfunctions

**Fig. 8:**
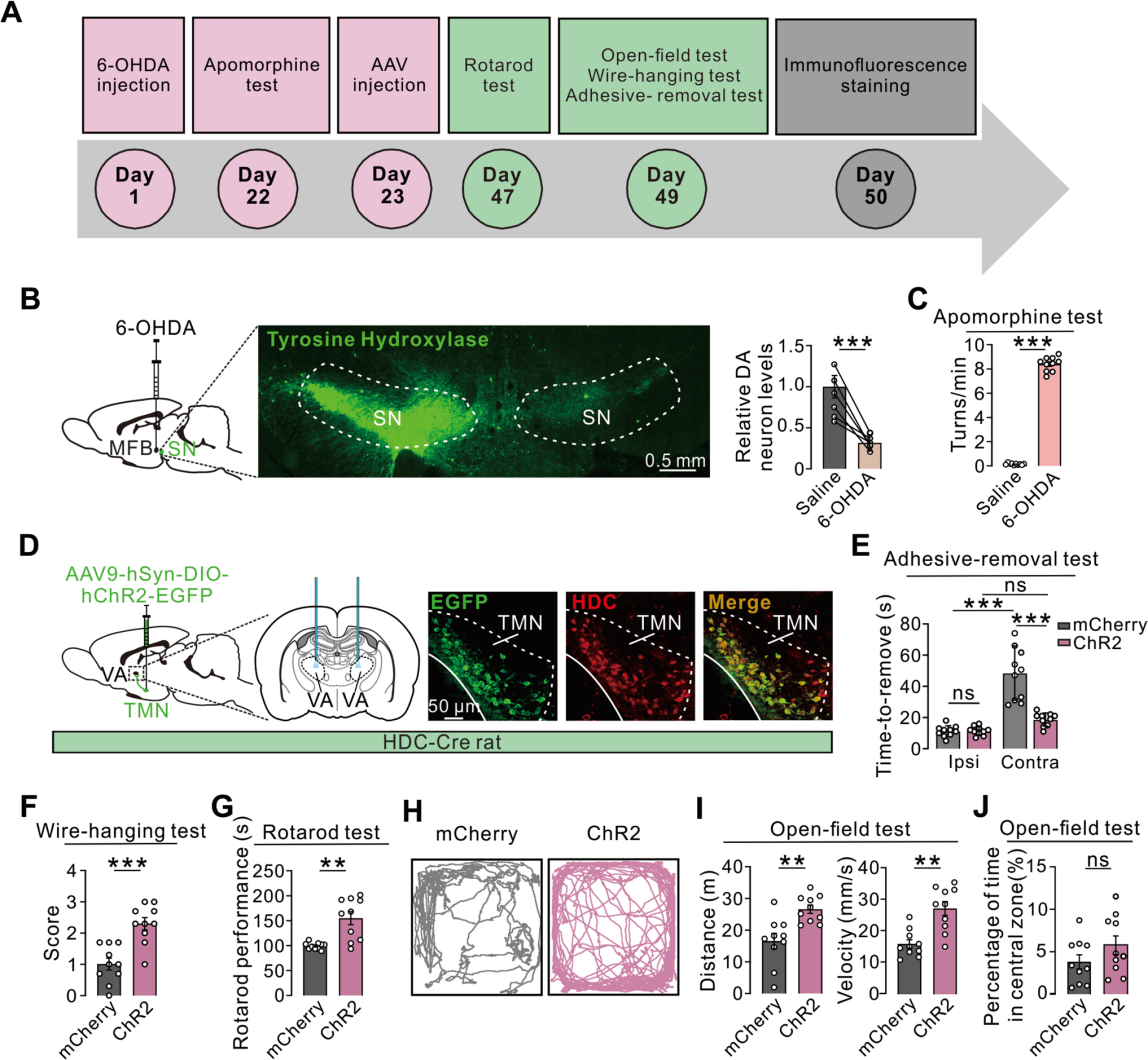
Optogenetic activation of histaminergic afferents in the VA ameliorates Parkinsonian motor dysfunction. **A** Timeline of the behavioral tests. **B** The population of TH-positive dopaminergic neurons in the ipsilesional SNc is significantly reduced in 6-OHDA-lesioned rats (*n *= 6 rats, *P *= 0.0007). **C** 6-OHDA-lesioned rats exhibit pronounced contralateral circling upon intraperitoneal administration of apomorphine (*n *= 10 rats, *P *< 0.0001). **D** ChR2-mCherry is selectively transduced in histaminergic TMN c neurons in HDC-Cre rats. **E** Optogenetic activation of histaminergic TMN-VA projections significantly shortens the prolonged contralesional adhesive-removal time in PD rats (*n *= 10 rats, *P < *0.0001), and restores the contralesional adhesive-removal time to the level of the ipsilateral side (*n *= 10 rats, *P *= 0.0533). **F** Optogenetic activation of histaminergic afferents in the VA remarkably increase the performance of PD rats in the wire-hanging test (*n *= 10 rats, *P *= 0.0004). **G** Optogenetic activation of histaminergic TMN-VA projections greatly extend the time for PD rats to fall off the rotarod (*n *= 10 rats, *P *= 0.0014). **H–J** Optogenetic activation of histaminergic TMN-VA projections increases the total locomotor distance (*n *= 10, *P *= 0.0014) and velocity (*n *= 10, *P *= 0.0011) of 6-OHDA-lesioned PD rats in an open field (I), but does not change the percentage of time spent in the central zone (*n *= 10, *P *= 0.2176) (**J**). Scale bars, 0.5 mm (**B**), 50 μm (**D**). Data are shown as the mean ± SEM. ns, no significant difference. *P < 0.05, **P < 0.01, ***P < 0.001, paired two-tailed Student’s *t*-test (**B**) or unpaired two-tailed Student’s *t*-test (**C**, **E**, **F**, **G**, **I**, **J**).

To determine the role of histaminergic afferents in the VA in motor deficits in PD (Fig. 8A–C), we used HDC-Cre rats to selectively transduce ChR2-mCherry in histaminergic TMN neurons (Fig. 8D). Optogenetic activation of the terminals of histaminergic TMN-VA projections significantly shortened and even rescued the prolonged contralesional adhesive-removal time in the 6-OHDA-induced rat model of PD (Fig. 8E). Moreover, optoactivation of the histaminergic afferents in the VA in PD rats increased the wire-hanging performance (Fig. 8F), extended the latency to fall off the rotarod (Fig. 8G), and improved the total movement distance (Fig. 8H, I) and velocity (Fig. 8H, I) in the open field. However, the percentage of time PD rats spent in the central area was not altered by the optoactivation of histaminergic VA afferents (Fig. 8J). Therefore, similar to the excitation of VA neurons, selective activation of histaminergic VA afferents also alleviated motor deficits but did not influence anxiety-related behaviors in PD rats. These results strongly suggest that histaminergic afferents may effectively ameliorate Parkinsonian motor dysfunction by activating VA neurons.

## Discussion

Although the VA of the thalamus holds a key position in relaying the outputs of the basal ganglia to the motor areas of the cerebral cortex [1, 3], its neuromodulatory afferents and their roles in the modulation of VA neuronal activity and related motor functions remain largely unknown. The present study reveals a lower baseline activity of VA in PD patients than in healthy subjects. In the 6-OHDA-lesioned rats, histamine induces a direct postsynaptic excitation of VA neurons by opening HCN channels coupled to H1 and H2 receptors and closing Kir and SK channels coupled to H1 and H2 receptors, respectively. Furthermore, histamine facilitated the post-inhibitory rebound excitation of VA neurons *via* the activation of HCN channels. The histaminergic VA afferent inputs not only promote physiological motor performance but also ameliorate Parkinsonian motor dysfunctions.

Histamine and histamine receptors in the central nervous system have been implicated in regulating various basic physiological functions, including motor control [11, 41]. In clinical practice, histamine-related agents have also traditionally been used to treat motor diseases such as vestibular disorders [42]. In the present study, we reveal, for the first time, that histamine postsynaptically excites the neurons of the VA, a critical area of the motor thalamus that transmits information from the basal ganglia to the cerebral cortex (Fig. 3). It is well known that H1 and H2 receptors are major postsynaptic histamine receptor subtypes expressed in the brain [43, 44], whereas H3 and H4 receptors are distributed predominantly in presynaptic terminals [13, 45] and peripheral immune cells [46, 47], respectively. The present result that the histamine-induced excitatory effect was blocked by H1 and H2 receptor antagonists (Fig. 4) suggests that H1 and H2 rather than H3 and H4 receptors mediate the effect of histaminergic VA afferents. Blockade of either H1 or H2 receptors in the VA significantly weakens motor initiation and other motor performance (Fig. 7), indicating an active role of endogenous histamine released by direct hypothalamic afferents in the VA in motor control. Notably, previous studies from our and other laboratories have reported that brain histamine improves motor initiation, motor coordination, and vestibulospinal reflexes as well as locomotion *via* their extensive impact on subcortical motor structures, including the basal ganglia [11, 48–50], cerebellum [51–53], brainstem vestibular nuclear complex [22, 53, 54] and spinal cord [55–57]. We thus suggest that central histamine acts as a general modulator for the motor system to maintain normal motor function.

Recent clinical findings have indicated that, in addition to regulating normal motor functions, the central histaminergic system is closely associated with PD. In PD patients, the concentration of histamine in the basal ganglia, including the substantia nigra, putamen, and globus pallidus, has been reported to be significantly increased [10]. Moreover, the histamine level in blood and histamine metabolites in cerebrospinal fluid has been reported to be highly positively correlated with the severity of PD [58, 59]. In addition, the density of histaminergic fibers and histamine receptors in the SN have also been found to be elevated in PD patients [10, 60]. Alterations of histamine homeostasis in the SNc have been shown to be associated with the risk for PD [61]. Since accumulating evidence suggests the involvement of histamine in the pathophysiology of PD, whether it is an attempt to compensate for dysfunction or merely an unimportant side-effect of disease progression has attracted increasing attention. In the present study, we reveal that activation of histaminergic fibers in the VA significantly alleviates motor deficits in PD rats (Fig. 8). Notably, optogenetic activation of VA neurons or TMN-VA histaminergic projections restored the performance of the adhesive-removal test in the contralateral forelimb to the control level of the ipsilateral side in a hemi-parkinsonian rat model (Figs 2F, 8E). Besides, our recent study has indicated that histamine can regularize the firing patterns of STN neurons, and DBS can increase histamine release in the STN and ameliorate Parkinsonian motor deficits in the 6-OHDA-induced rat model of PD [11]. Moreover, histamine has been reported to act on the entopeduncular nucleus to ameliorate Parkinsonian motor symptoms [48]. Therefore, the present results, together with previous studies from our [11, 62] and other laboratories [48], suggest that increased histamine may be a compensatory mechanism for basal ganglia dysfunction and motor deficits. In addition to motor symptoms, PD patients manifest non-motor phenotypes, such as anxiety, sleep disturbance, and cognitive impairment [32, 33]. In the present study, both direct excitation of VA neurons and activation of histaminergic VA inputs had no impact on anxiety behaviors in PD rats (Figs 2K, 8J). Although further studies are needed to determine whether histaminergic VA input is involved in other nonmotor PD symptoms, the fact that the VA constitutes a major interface between the basal ganglia and the motor areas of the cerebral cortex suggests that histamine is involved in the motor rather than the non-motor symptoms of PD.

The effect of histamine on VA neuronal activity provides a mechanism for why activation of histamine afferent inputs alleviates PD motor deficits. However, there is still no consensus on whether the firing frequency or firing pattern of thalamic VA neurons plays a more important role in PD [1, 3, 63]. In our study, a lower baseline activity in the VA in PD patients was found by resting-state fMRI (Fig. 1), which is in line with the results of neuronal recordings during microelectrode-guided functional stereotactic neurosurgery in PD patients [4]. Moreover, optogenetic excitation of VA neurons or histaminergic afferent terminals effectively alleviates motor deficits in the 6-OHDA-lesioned HDC-Cre rat model of PD (Figs 2F–J, 8E–I), suggesting that increasing the firing rates of VA neurons alone may improve PD motor function. Other studies also indicate that burst firing of VA neurons may be involved in the motor symptoms of PD, which can be decreased by clinically effective DBS of the STN or globus pallidus externa [64, 65]. Firing synchrony is another factor involved in PD. In dopamine-depleted non-human primates, synchronized oscillations of VA neurons increase before parkinsonism becomes evident [66]. Interestingly, we also found that histamine promotes the rebound firing of VA neurons *via* the activation of HCN channels coupled to H1 and H2 receptors (Fig. 5C–F). Whether the alteration of firing patterns of VA neurons may also contribute to the histamine-induced amelioration of PD motor symptoms remains to be further explored.

In conclusion, our results demonstrate that baseline activity in the thalamic VA is decreased in PD patients, and histamine ameliorates PD motor deficits in the 6-OHDA rat model *via* exciting VA neurons through the activation of HCN/Kir channels coupled to H1 receptors and HCN/SK channels coupled to H2 receptors. These findings not only contribute to understanding the role of the VA and the central histaminergic system in basal ganglia motor function and PD motor dysfunction but also provide insight into clinical treatment strategies for PD by targeting the histaminergic system and the ion channels coupled to histamine receptors. It is important to note that there are still gaps between the fundamental research on the histaminergic VA afferent system in the 6-OHDA-induced rat model and clinical therapeutic strategies for PD treatment. Firstly, although the 6-OHDA-induced rat is the most extensively used rodent model of PD, no deposition of Lewy bodies, the pathological hallmark in PD patients, has been reported in them. Therefore, whether targeting histamine receptors can alleviate the motor symptoms in MPTP primate models of PD may be a key critical step in the translational process. Secondly, considering that PD is 1.5 times more frequent in men than women [67], only male rats have been focused on in the present study. However, whether there is a sex difference in the amelioration of PD motor deficits by activation of histaminergic VA inputs remains unknown. Finally, how to selectively and efficiently activate VA histamine receptors is still a challenge. Targeted drug delivery of histaminergic agents or transcranial-focused ultrasound neuromodulation that directly activates VA neurons may be promising treatments for PD in the future.

## Data Availability

The data that support the findings of this study are available from the corresponding author upon reasonable request.
